# A Manualized Behavioral Therapy Intervention for Youth with Autism Spectrum Disorder and Substance Use Disorder

**DOI:** 10.1155/2023/8998160

**Published:** 2023-04-01

**Authors:** James McKowen, Amy Berger, Joshua Towbin, Amy M. Yule, Diana Woodward, Lisa Nowinski, Gina Forchelli, Robert J. Meyers, Gagan Joshi, Timothy E. Wilens

**Affiliations:** ^1^Clinical and Research Programs in Pediatric Psychopharmacology and Adult ADHD, Massachusetts General Hospital, 55 Fruit Street, Boston, MA 02114, USA; ^2^Department of Psychiatry, Boston University School of Medicine, Boston Medical Center, 720 Harrison Avenue, Suite 915, Boston, MA 02118, USA; ^3^Lurie Center for Autism, Massachusetts General Hospital, 1 Magurie Road, Lexington, MA 02421, USA; ^4^Center of Alcoholism, Substance Abuse and Addiction, University of New Mexico, 2650 Yale Blvd., NM 87106, USA

## Abstract

Research highlights the increasing overlap of autism spectrum disorder and substance use disorders in young people. However, no behavioral treatments exist addressing this comorbidity despite great need. A team of clinicians developed an integrated behavioral protocol addressing substance use in youth with autism spectrum disorder. The multidisciplinary team developed 12 youth, 7 parent, and 3 joint modules based on established evidence-based therapies shown to have effectiveness separately addressing autism spectrum and substance use. Two cases are discussed to illuminate this integrated intervention. Adaptations to the protocol were made during feedback from patients and their parents. Further research is needed to determine the effectiveness of this preliminary protocol.

## 1. Introduction

Autism spectrum disorder (ASD) is a neurodevelopmental disorder characterized by difficulties with social communication and interaction, as well as repetitive patterns of behavior and includes social communication disorder on the spectrum [1]. It is estimated that prevalence of ASD is approximately one in forty-four children 8 years and older [2]. ASD is associated with significant disability including dependence on family members through adulthood [3], reliance on social services [3], and lack of employment [4]. Individuals with ASD frequently have difficulties with social communication [1] and often with emotion regulation [5].

It is well documented that psychiatric comorbidities are common in ASD [6–8]. For example, one meta-analysis found that the rates of the three most common psychiatric disorders comorbid in a referred ASD population were 28%, 20%, and 12-13% for attention-deficit hyperactivity disorder (ADHD), anxiety disorders, and disruptive behavioral disorders, respectively [9]. Less is known, however, about comorbid ASD and substance use disorders (SUD) [10–12]. Recent research has yielded inconsistent findings on the prevalence of SUD in adults with ASD [13–15] with rates ranging from 1 to 36% reported. This large variability likely reflects the varied samples and methodologies used assessing SUD. Despite the lack of definitive data on substance use in people with ASD, it has been recently reported that 20% of young people aged 16-26 years old presenting for primary SUD treatment manifest prominent ASD traits as assessed by parental report on the social responsiveness scale–second edition (SRS-2) [16]. Althougth the SRS-2 is not an autism diagnostic tool, the results of this study suggest that when present, elevated autism traits by parental report appear to signal a distinct subset of clients (see McKowen et al. [16] for additional information).

Research examining the comorbidity of ASD and SUD is critical given that the majority of SUD programs do not routinely assess for the presence of developmental disorders such as ASD [14], nor do any behavioral interventions exist specifically targeting this complex population. Thus, patients with ASD and SUD are left both underidentified and undertreated. This point was highlighted by Regnit and colleagues [17] who in a case example described the need to address substance use in those with autism [17]. They noted the need to address ASD behaviors such as rigidity, perseveration, communication challenges, and comfort seeking along during SUD treatment. Likewise, considerations in the management of ASD and SUD including communication difficulties, varied capacity for motivation to change behavior, and different impacts of social influence have been noted in prior papers [10].

To date, only two studies have examined the utility of evidence-based Cognitive Behavioral Therapy (CBT) in the treatment of adults with SUD and ASD. First, Helverschou et al. [18] in a very small sample of four cases provided CBT-informed supervision to clinicians treating adults with ASD and SUD. Results suggested two participants ended their substance use, one reduced their use, and one continued to heavily drink alcohol. Second, Walhout et al. examined a CBT group-based treatment in adults with ASD and SUD [19]. They found improvements in alcohol use, depression, anxiety, and use of active coping and social support coping but no changes in other core challenges common to ASD such as rumination.

Despite the two aforementioned studies, no protocols focused on individual therapy have been developed or published, and nothing in youth. We thus developed the first manualized behavioral intervention integrating three empirically based treatments. We describe the development of this protocol and review two cases with ASD traits and SUD who received the intervention as part of routine clinical care for SUD.

A multidisciplinary team of board certified licensed clinicians with extensive experience researching and treating those with autism, substance use, or both, met over the course of one year to develop a protocol for treating comorbid ASD and SUD. Based on established literature and the teams experience, the following treatment approaches were decided upon as optimal to be integrated into a protocol addressing the comorbidity of ASD and SUD in youth: Cognitive Behavioral Therapy (CBT), Adolescent Community Reinforcement Approach (A-CRA), Social Skills Training (SST), Dialectical Behavioral Therapy (DBT), and Community Reinforcement and Family Training (CRAFT; for the parent protocol specifically). All these approaches have demonstrated evidence supporting their treatment of ASD and/or SUD yet none have been examined in comorbid ASD + SUD.

More specifically, CBT is a well-established treatment for substance use, both in individual and group modalities [20], and has been shown to address anxiety in [21] and emotion dysregulation broadly [22] in those with ASD. A-CRA is a treatment approach developed by Substance Abuse and Mental Health Services Administration (SAMHSA) to treat youth with SUD aged 12 to 24 years old. This approach is centered around helping individuals identify their goals using the Happiness Scale, develop an understanding of their use patterns using functional analysis of triggers, and expand prosocial behaviors to replace activities related to substance use through conducting a functional analysis of substance use [23]. Harm reduction has been cited as likely being more effective in those with ASD also regarding certain challenging behaviors [24, 25]. ACRA has not been studied in those with ASD. SST is widley used in populations with ASD, primarily to improve social skills through participant modeling, social problem solving, and self-monitoring [26], and some studies have shown that social skills training may help reduce adolescent substance use [27]. Given the core aspect of skills deficit in those with ASD, it was agreed that this should part of this protocol. DBT is a treatment that focuses on teaching patients how to cope with and change intense emotions and unhealthy behaviors [28]. DBT has been shown to be effective in addressing addiction [29, 30] and has been modified to treat adults with ASD. Specifically, all four modules of DBT skills training (distress tolerance, interpersonal effectiveness, mindfulness, and emotion regulation) have been examined in both addiction and ASD, but not in those with both ASD + SUD [31, 32]. Given the commonality of distress intolerance in those with ASD as well as SUD, the module of Distress Tolerance (DT) was chosen to be included. Indeed, DT as a standalone module in those with addiction has shown benefit [33]. Finally, for the parents, CRAFT was developed by Meyers et al. [34] to target concerned significant others of individuals with SUD, including parents of youth. This approach emphasizes contingency management training, communication skills, and planning activities to compete with substance use to allow concerned family and friends to improve their relationships with individuals with substance use and ultimately reduce their loved one's substance use [34]. A large part of CRAFT is also addressing caregiver stress through self-care planning. In a pilot study, CRAFT has also shown utility in improving well-being of parents of adults with ASD [35].

The protocol consists of 12 youth modules, 7 parent modules, and 3 joint modules. The team elected an individual therapy model versus the common group-based care seen in outpatient SUD programs due to the goals of integrating parents into care in parallel and in joint sessions. Separate providers meet with the youth and the parents to maintain alliance and boundaries. This protocol was developed specifically for young people aged 26 years and under given the lack of protocols in youth. Session length was proposed to be 45-50 minutes with sessions weekly.

Table 1 outlines the specific modules within the youth module. The practitioner begins treatment by establishing a rapport with the patient, educating the patient on characteristics of ASD and SUD, and establishing goals for the treatment using the ACRA Happiness Scale. As the sessions continue, the patient practices identifying their emotions (adapted from CBT), analyzing the circumstances that lead to their substance use (adapted from ACRA functional analysis of triggers), and establishing healthy behaviors that they can do to replace substance use such as behavior activation and alternative coping strategies (adapted from both ACRA and CBT). The patient then practices abstinence from substances (adapted from ACRA sobriety sampling module), effective social-communication skills (adapted from SST), emotion regulation (DT module from DBT), and anxiety management (adapted from CBT).

In the parent modules, parent(s) meet with a separate practitioner over the course of seven weeks starting at the same time as youth sessions. These sessions begin with the parent explaining their concerns about their child and learning about characteristics of ASD and SUD. As the sessions continue, the parent learns about contingency management and functional analyses of their child's substance use and other problem behaviors (adapted from CRAFT). The parent sessions conclude by learning the strategies addressed in the youth modules such as effective communication, problem solving, emotion regulation, and anxiety management skills their child is learning. In these sessions, the parent also focuses on self-care and developing a safety plan for their child. See Table 2 for a summary of the parent modules.

In the joint sessions, which can be administered flexibly as needed but ideally after both parent and youth have completed 4-5 sessions so as to get a sense of the challenges and goals within the youth-parent system, the parent and youth meet together along with both practitioners in a group format. In these sessions, the parent and child work to develop a shared understanding of their symptoms of ASD and the context of their substance use. They also practice effective communication and healthy behaviors to replace substance use as well as review any contingency supporting change. See Table 3 for a summary of the joint sessions.

## 2. Case Presentations

All clinical information regarding the patients who completed this intervention has been deidentified to protect the privacy of these individuals. This protocol development was IRB exempt as this was not a study, and case reports are not considered research. Therapists were licensed in psychology or social work with at least 5-10 years of clinical experience in working with youth in SUD and/or ASD. Both providers were part of the protocol development team. Patients were initially evaluated at treatment entry using the GAIN [36] and standardized clinical assessment questions following DSM-V diagnostic criteria [37]. Assessment of substance use was done by asking frequency of use at intake and between each session, i.e., number of days/week of substance use. As this case report details cases from standard clinical practice, self-report rating scales assessing substance use and autism were not used; however, the Clinical Global Impressions Scale-Severity (CGI-S) and -Improvement (CGI-I) scores were used to assess severity of both ASD and SUD at baseline, and then, at end of treatment, the CGI-I was used to assess gains made. The CGI is commonly used by nonresearcher clinicians to rapidly assess treatment response and progress [38]. Treatment took place within an academic medical center at an outpatient substance use treatment program specializing in youth addiction care. To date, the protocol has been implemented for approximately 14 months.

### 2.1. Case A

Youth patient A (YP-A) was a 17.5-year-old, Caucasian male with previous diagnosis of ASD from neuropsychological testing at age 8. At intake, he reported history of heavy binge use of alcohol (approximately 2 binge episodes per week, 6-8 standard units of alcohol per binge) and marijuana (5/7 days of cannabis use, unknown potency, but 1-2 joints per time). He had also been misusing cough medications daily (approximately 900 mg of Delsym; dextromethorphan per occasion) for the majority of days in the three months prior to the initial evaluation and reported stealing this product from a local pharmacy. YP-A and his parents also reported challenges in patient's emotional regulation typified by explosive outbursts with intermittent nonsuicidal self-injurious behavior (NSSIB) in the home. He was diagnosed with DSM-V autism level 1, alcohol use disorder – mild and cannabis use disorder – mild. He did not meet DSM-V criteria for other substance use despite evidencing problematic use patterns, a rule out of mood disorder was also given due to explosive behavior and intermittent NSSIB. Age of onset of first alcohol use was 16 years, cannabis 16 years, and dextromethorphan was 17 years. All DSM-V substance use diagnoses were given at the intake with assessed time period being the past 12 months. Family history was significant for alcohol problems on paternal side (although it was not known who specifically). Treatment goals focused on safety planning, communication skills, functional analysis of use, contingency management, and emotion regulation skill development.

YP-A was able to complete 9/12 youth modules (75% of modules implemented) over 11 sessions, over approximately a 5-month period; specifically, those focusing on psychoeducation about ASD and SUD, emotional identification and regulation skills training, sobriety sampling, and social and communication skills. The order of modules was altered at times to accommodate the patient's needs of the session, and modifications were made to abbreviate some modules or extend them over multiple sessions as the patient was only able to tolerate meetings that were less than 20 minutes in duration. The patient was able to track use of substances, set goals for reducing use of cannabis, avoid binge drinking, and set a 30-day sobriety sample from more risky cough syrup use. He also used emotion regulation skills and communication strategies to reduce anger outburst with his parents at home. The patient was also willing to engage with the psychiatrist for a trial of mood stabilizing medication by seeing the clinic psychiatrist.

Parent engagement was limited as his parents were well educated on ASD given the early diagnosis in childhood. Parents completed 5/7 modules (70% of modules implemented) with sessions focused on how to use contingency management and communication skills in order to enhance positive reward for improved or nonsubstance use behavior, reduced behavioral dysregulation (e.g., anger management–taking space, using communication skills more effectively, reaching out to therapist when in distress) as well as improving overall relationships within the home. A joint session via telehealth was unsuccessful due to YP-A feeling overwhelmed with the video-based format (implemented during the COVID-19 pandemic; thus, a combination of video visits were done) Despite limited parental engagement, this patient engaged consistently with therapy, started medication to target his mood, and was still smoking cannabis but fewer times per week (3-4 days per week). Importantly, he had not used cough medicine or engaged in binge alcohol use since starting treatment. Though not sober, engaging in less risky substance use was an important treatment outcome in this case. Regarding CGI outcomes incorporating both patient and parent therapist impressions, outcomes for substance use, YP-A initial CGI-S score was 5-6 (markedly/severely ill), and at the end 3 (mildly ill), with CGI-I indicating 2 (much improved). Regarding ASD symptoms, initial CGI-S score was 4 (moderately ill), and at the end 3 (mildly ill), with CGI-I indicating 2-3 (much improved/minimally improved).

### 2.2. Case B

Youth patient B (YP-B) was a 17-year-old, Caucasian, male with a DSM-V diagnosis of social communication disorder based on prior neuropsychological assessment at age (date of diagnosis unknown). He was assessed at intake and reported problematic use of both cannabis (approximately 4-5 days/per week, unknown potency, but 3-4 “hits” per day) and alcohol (binge drinking approximately 1-2 days per week, 3-4 standard alcohol units per occasion) but met criteria for DSM-V cannabis use disorder, but none for alcohol use disorder. Age of onset of first alcohol use was 15 years, and cannabis was age 14 years. All DSM-V substance use diagnoses were given at the intake with assessed time period being the past 12 months. He also reported history of anxiety consistent with a diagnosis of Anxiety Not Otherwise Specified. Family history was significant for two maternal uncles with substance use disorder. Treatment goals focused on reducing risky substance use to prevent a worsening course as well as improving anxiety management and general social skill challenges noted by him and his parents such as being influenced to use by peers. YP-B completed 11/12 modules over 14 sessions (90% of modules implemented), over a 6-month period of time. In addition, two joint sessions were held to review communication strategies, ASD concepts, and contingency management to support lower cannabis smoking.

YP-B's parents were less familiar with this patient's diagnosis of social communication disorder and their impact on his presentation given he was a relatively high functioning senior in high school. Parents covered 6/7 modules (85% of modules implemented) including psychoeducation on ASD, communication challenges, and strategies to support his anxiety related to social engagements and changes in routine. The contingency management module was also utilized, incentivizing negative toxicology screens tied to car driving privileges. During the duration of treatment, the patient reduced his cannabis use by approximately 50% fewer use occasions (1-2 days per week instead of four, 1-2 hits per time), did not engage in a binge drinking episode, and felt less anxious at school which also allowed him to successfully transition to a summer school enrichment program to bolster self-esteem and leadership skills. Regarding CGI outcomes incorporating both patient and parent therapist impressions, YP-A initial CGI-S score was 3 (mildly ill) and at the end 2 (minimally ill), with CGI-I indicating 2 (much improved). For ASD symptoms, initial CGI-S score was 3 (minimally ill), and at the end 2-3 (borderline/minimally ill), with CGI-I indicating 3 (minimally improved).

## 3. Discussion

This report describes the development of an integrated, nonproprietary, flexible manualized behavioral therapy derived from empirically based therapies previously shown separately to evidence improvement in ASD and SUD but integrated here to address both. An initial description of two clinical cases treated with this protocol exemplifies its implementation within a routine clinical outpatient setting. Both cases discussed here evidenced improvements in CGI-S and CGI-I in substance use at the conclusion of the protocol implementation. Less change was noted in ASD symptoms, which is not surprising given the relatively short intervention period and more refractory nature of ASD.

Flexibility in the delivery of the protocol was a key aspect in the development of the intervention. For example, for YP-A, sessions were modified to be briefer, more specific, and include handouts to enhance structure and provide concrete information to assist in the engagement process with treatment. Given varied familiarity with ASD and social communication disorders, alteration in the degree of psychoeducation delivery to patients and their families was necessary. For example, the parents of YP-A were more familiar with ASD and needed less education about its manifestation. Integration of socially relevant topics such as friendship quality, exposure to risks of being “taken advantage of,” a common risk for youth with social communication deficits and SUD [24], were also made. Indeed, YP-B was easily influenced by peers and learning communication skills, and boundaries were helpful in learning to navigate this. Given the common experience of elevated anxiety in those with ASD [39], and anger in those using cannabis [40], modules addressing affect management through CBT and DBT were particularly emphasized. Indeed, both YP-A and YP-B struggled with mood and anxiety issues, respectively, and both used substances to self-medicate; therefore, these modules were particularly relevant to their learning more effective coping strategies. Indeed, it has been speculated that individuals with ASD may use more substances to manage heightened affective distress and social rejection [41, 42].

Our protocol also is aimed at explicitly including parents given that parenting youth with both ASD and SUD is particularly challenging. Young people with SUD have been found to be more dependent on family members over time, and this may be particularly of relevance in context to ASD [24, 43, 44]. Hence, family members living with young people with both disorders may struggle to support their family member towards independence and adaptive developmental functioning (e.g., remembering to use specific therapy skills, making appointments, budgeting etc..). Thus, incorporating family into treatment is critical, yet often neglected in behavioral therapies of young adults. Of the two sets of parents discussed previously, both found the opportunity to have an intervention dedicated to support their parenting instrumental in learning adaptive approaches. Our observations support the modules related to psychoeducation, contingency management, communication, and teaching parents the skills their child is learning to be most valuable. Discussion of parental self-care and wellness throughout the intervention was also particularly well received.

Generally, for clinicians working with those with ASD and SUD, enhanced training on screening, assessment, and intervention is critical given that most adult providers receive little to no training in developmental disorders such as ASD [24]. Moreover, given the relatively high rate of ASD traits in young people with SUD, further training to understand the manifestation of ASD may be particularly helpful. Patients with ASD may be formulated as unmotivated for care as they are often lost, late, rigid to change, and can be socially challenging in group therapy in the absence of understanding their underlying vulnerabilities. Conceptualizing these behaviors within an ASD framework can enhance empathy and understanding and provide more tailored SUD treatment where it is greatly needed. In support of this, Helverschou and colleagues have preliminarily shown that providing clinicians who work with clients with ASD and SUD supervision embedded in evidence-based treatments such as CBT even in the absence of a tailored protocol may be effective for SUD [18].

Despite the important initial contribution of this work, there are a number of substantial limitations needing discussion. First, this protocol was used as part of routine clinical care in only two patients and their parents. Although beyond the scope of this paper, this very small sample limits generalizability as no quantitative scales measuring SUD or ASD were implemented as part of this clinic's routine and hence not presented. However, these two cases are not atypical of common presentations of ASD (and spectrum including social communication disorder) and coexisting SUD, and we did include CGI-S and CGI-I as proxy outcomes summing patient and parent therapist impressions. Second, given the goal to limit the number of sessions to be within a reasonably circumscribed amount, we did not extensively cover topic areas such as executive functioning strategies, Internet/gaming compulsive use, legal challenges, job training, how to engage with support services, or how to access social services. This maybe in part why we saw less improvement in ASD symptoms compared to SUD, and thus, future examination of topics is indicated. Third, we did not explicitly address issues around race, ethnicity, gender, gender identity, or sexual orientation in this iteration. These topics would warrant consideration for more explicit discussion. Finally, while we designed this protocol to be flexible in its administration, our two cases reports and heterogeneity in administration did not allow us to speculate on which components were the most useful. In each case presented, although not all modules were implemented, approximately 70% or more of modules were implemented across the youth and parent modules. Seventy percent at minimum may be an important threshold to meet to ensure fidelity to the protocol—further research is needed in that area however to determine what threshold is considered acceptable within a flexible modular intervention.

Despite these limitations, to our knowledge, this is the first behavioral therapy protocol designed for young people with the comorbidity of ASD/significantly impairing ASD traits and SUD. Both cases evidenced reductions in substance use and overall improvement in global functioning. Clearly however, much more substantial research is needed within a more diverse, larger pool of patients and their families incorporating standardized assessment measures of improvements.

## Figures and Tables

**Table 1:** Protocol content of youth modules for youth with autism spectrum disorder (ASD) and substance use disorder (SUD).

Module	Content	Exercises
1	Introduction and goals	Happiness scale
		Goals of counseling form
		Making a safety box worksheet
		Making a crisis plan worksheet

2	Psychoeducation	Review diagnostic criteria of ASD and SUD

3	Emotion identification	Assessing emotion identification questionnaire
		Sorting your thoughts, emotions, and behaviors worksheet
		Identify triggers worksheet

4	Functional analysis of substance use behavior	Functional analysis of substance use behavior form

5	Changing use	Early warning system
		Refusal training
		Cognitive restructuring

6	Functional analysis of healthy activities	Functional analysis of healthy activities
		Review healthy activities

7	Sobriety sampling	Set goal for abstinence trial
		Discuss plan to remain abstinent
		Develop a back-up plan

8	Social and communication skills	Role play exchanges
		Thinking about your current relationships worksheet
		Increasing the relationships in your life worksheet

9	Problem solving	How can I solve this problem worksheet

10	Emotion regulation	Mindfulness practice
		Distracting skills worksheet
		Grounding techniques

11	Anxiety management	Sorting out your thoughts, emotions, and behaviors grid
		Unhelpful thinking styles sheet

12	Treatment closure	Reflect on skills learned
		Discuss what was most useful
		Discuss next steps/aftercare

**Table 2:** Protocol content of parent modules for parents of youth with autism spectrum disorder (ASD) and substance use disorder (SUD).

Module	Content	Exercises
1	Introduction and goals	Obtain history of presenting problem
		Discuss parent's role in treatment
		Discuss reinforcers for change
		Parent self-care and safety

2	Psychoeducation	Review diagnostic criteria of ASD and SUD
		Emotion identification activities

3	Contingency management	Discuss internal and external motivators for change
		Review efficacy of contingency management
		Contingency management fact sheet

4	Functional analysis of substance use behavior	Functional analysis of substance use behavior form
		Review abstinence vs. harm reduction
		Discuss short-term reinforcers of behavior
		Functional analysis of healthy activities form

5	Communication skills and problem solving	Communication skill worksheet
		Role play effective communication
		How do I solve that problem worksheet
		How can I solve that problem worksheet

6	Emotion regulation and anxiety management	Discuss emotion regulation and anxiety management
		Highlight challenges within diagnosis of ASD

7	Relapse prevention and treatment closure	Review goals of treatment
		Discuss parent perception of progress
		Discuss continuing care
		Review warning signs for relapse

**Table 3:** Protocol content of joint modules youth with autism spectrum disorder (ASD) and substance use disorder (SUD), and their parents.

Module	Content	Exercises
1	Psychoeducation and skills review	Develop shared understanding of symptoms of ASD and context of substance use
		Ask parent to share concerns and hopes around improvement in SUD in their child
		Review functional analysis of substance use
		Discuss strategies to manage cravings and improved emotion management

2	Communication and problem solving	Review communication skills
		Problem solving procedure

3	Functional analysis of healthy activities	Discuss importance of identifying a healthy activity as an alternative to substance use
		Set goals around implementing the activity

## Data Availability

All conclusions were drawn from descriptive chart reviews included in this manuscript. No additional data was used to support the conclusions of this study.
